# Cutaneous Reactive Lymphoid Proliferation Arising in the Setting of Concomitant Antidepressant and Antihypertensive Therapy

**DOI:** 10.7759/cureus.60681

**Published:** 2024-05-20

**Authors:** Taylor Fleshman, Shane Cook

**Affiliations:** 1 Dermatology, Marshall University Joan C. Edwards School of Medicine, Huntington, USA

**Keywords:** reactive lymphoid hyperplasia, cutaneous lymphoid hyperplasia, lymphocytoma cutis, cutaneous pseudolymphoma, pseudolymphoma

## Abstract

Cutaneous reactive lymphoid proliferation (CRLP) is a condition that resembles cutaneous lymphoma, and differentiating the two is necessary for proper diagnosis and treatment. It can be idiopathic or caused by viruses, drugs, or skin trauma, resulting in reactive lymphoid hyperplasia. Several clinical and histopathological features are helpful for differentiating CRLP from lymphoma, and they must be considered as a whole to reach the correct diagnosis. The number, location, and progression of CRLP lesions are important clinical clues, while the type, size, arrangement, surface markers, and clonality of the cellular infiltrate are key histopathological clues. We present a case in which CRLP arose in the setting of concomitant antidepressant and antihypertensive use, which are both potential causes of CRLP. In this case, excision served as both diagnosis and treatment. The benign presentation and lack of clonality led to the diagnosis of CRLP. While the cause is unknown, drug exposure was a possible inciting factor, and the patient will be monitored for recurrence.

## Introduction

Cutaneous reactive lymphoid proliferation (CRLP), formerly called cutaneous pseudolymphoma, is a benign, reactive condition involving collections of lymphocytes in the dermis, which may resemble lymphoma clinically and/or histologically [1]. CRLP may be classified by clinical or histopathological presentation, the type of infiltrate, or the inciting agent [2]. The infiltrate may be predominately T-cell, B-cell, or a mixture and is usually polyclonal [2]. Known causative agents include skin trauma, arthropod bites, viruses, spirochete infection, tattoo dye, injections, and certain drugs, but it also may be idiopathic [1-2]. Over a quarter of CRLP cases are caused by drug exposure [3]. Common causes of drug-induced CRLP are anticonvulsants, antidepressants, antihypertensives, and monoclonal antibodies [4-5]. The most frequent presentation is a solitary papule, nodule, or plaque, but others include multiple or disseminated lesions and even erythroderma [2, 6]. B-cell CRLP usually presents above the neck or on the upper thorax, and it is more common in those who are white, male, and under age 40 [1-2, 6]. Epidemiological differences are unknown for T-cell CRLP [1-2]. We present a case of nodular CRLP in the setting of concomitant antidepressant and antihypertensive use. The purpose of this case report is to highlight the key diagnostic features of CRLP and the pervasiveness of its triggers.

## Case presentation

**Figure 1:**
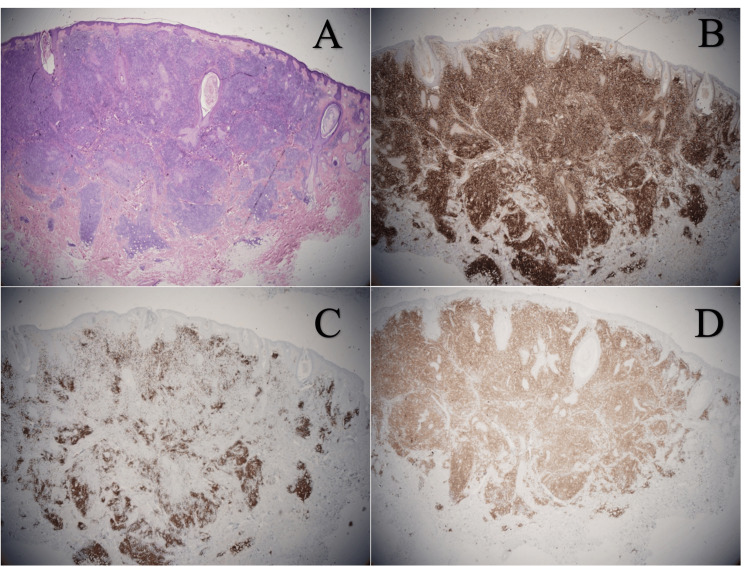
(A) Dense, nodular lymphocytic infiltrate in the dermis with germinal center formation; (B) Immunohistochemistry stain showing BCL2 positive cells forming reactive germinal centers; (C) Immunohistochemistry stain showing CD20 positive B cells; (D) Immunohistochemistry stain showing CD3 positive T cells

A male in his 30s presented as a new patient after a referral from urgent care for an irritated lesion on the face. It presented two months prior and had progressively grown. He did not report any bites or injections in the area. Failed attempts were made to drain its contents prior to presentation. Medical history included hypertension, acid reflux, anxiety, and depression. Medications at onset included vortioxetine, buspirone, lisinopril, omeprazole, diclofenac, and ergocalciferol. On examination, there was a 1.5 cm firm, erythematous nodule on the right cheek with no fluctuance or central pore. Additionally, there were papules on the nose that were consistent with rosacea. He had no lymphadenopathy, fever, or weight loss. The patient was given doxycycline (100 mg twice daily) with plans to excise if the nodule did not improve. The lesion was still painful the following week, so the patient returned for excision. The sample was sent for pathology and subsequent gene testing. The pathology report described a dense, nodular, lymphocytic infiltrate in the dermis with scattered plasma cells and focal germinal center formation consistent with reactive lymphoid hyperplasia (Figure 1A). Staining for BCL2, CD20, and CD3 revealed a mixed B- and T-cell lymphocytic infiltrate with well-formed germinal centers (Figure 1B-1D). The kappa to lambda ratio was normal, and gene studies showed no clonal immunoglobulin heavy chain (IgH) gene rearrangement. Based on these results, excision was deemed sufficient treatment. 

## Discussion

Diagnosis of CRLP requires careful clinical and histopathological correlation. Histological evaluation of the pattern, morphology, and characteristics of the infiltrate, as well as immunophenotypic and molecular studies, may assist differentiation from its malignant counterpart [7]. Testing for clonality and IgH gene rearrangement is helpful because malignant cases are generally monoclonal, but clinical context is necessary because there have been early cases of lymphoma that did not exhibit clonality and some cases of CRLP that did [2,7]. On histology, CRLP has highly proliferative germinal centers, tingible body macrophages, and scattered plasma cells, while malignant cases tend to have less active germinal centers, lack tingible body macrophages, and have more pronounced plasma cells [2,7]. In lymphoma, lymphocytes are more likely to be large and atypical, and they may lack markers CD2, CD3, CD5, and CD7 [1]. Clinically, small, localized lesions are more likely to be CRLP, while diffuse, large, or progressive lesions are worrisome for cutaneous lymphoma [1,7]. Under dermoscopy, a solitary nodule with an orange background, follicular plugging, and organized linear or arborizing vessels is suspicious for lymphoma or CRLP [8]. However, this only rules out other causes and does not help differentiate the two.

Treatment is determined by the etiology of the disease, with options including topical or intralesional corticosteroids, surgery, laser therapy, antibiotics, antivirals, and avoidance of the causative agent [3]. In diffuse or unrelenting disease, phototherapy and hydroxychloroquine are possible treatment options [3]. Generally, the disease does not progress, and some may even regress after biopsy; however, there have been cases of CRLP transforming into lymphoma [2, 9]. Some may persist for months or years, and cases caused by external factors may recur upon re-exposure [2].

The time between exposure and onset can vary from days to months or even years, with antidepressants having a shorter median time to onset [5]. Our patient was taking both vortioxetine and lisinopril when the lesion presented, which could be a potential etiology. Lisinopril was started several years prior, and the dose was increased four months prior to lesion onset. Vortioxetine was started five months prior to lesion onset and increased three months prior. Based on a score of four on the Naranjo scale for each drug, it is possible that this patient's CRLP was a drug reaction [10]. Since the nodule was symptomatic, excision was chosen over biopsy. Excision seems to be sufficient treatment as of now, and adjustment of medications is not warranted, but the patient will be monitored for recurrence.

## Conclusions

Differentiating CRLP from cutaneous lymphoma requires careful clinical and histopathological correlation. Biopsy with immunophenotyping and IgH gene rearrangement studies may help with diagnosis, but they are only one part of the clinical picture. Characteristics that may indicate lymphoma over CRLP include skin findings that are diffuse, progressive, or unremitting, with biopsy results showing pronounced plasma cells, a lack of tingible body macrophages, and monoclonality. Recalling that commonly prescribed drugs may present with CRLP may clarify the diagnosis, and prescribers should be aware of this association. Excision is often curative, but more conservative treatments include topical or intralesional steroids, antibiotics, and avoidance of the causative agent. In rare cases, CRLP may progress to cutaneous lymphoma, so patients who do not reach complete clearance should be monitored.
